# Advancing IHR capacities in the DRC: findings from the 2022 e-SPAR and NAPHS evaluation

**DOI:** 10.1093/inthealth/ihaf145

**Published:** 2026-01-13

**Authors:** Jean Paul Muambangu Milambo, Longo Mbenza Benjamin

**Affiliations:** Department of Obstetrics and Gynaecology, Faculty of Medicine and Health Sciences, Walter Sisulu University, Private Bag X1, Mthatha 5100, Eastern Cape, South Africa; Department of Industrial and Organisational Psychology, University of South Africa (UNISA), P.O. Box 392, Pretoria, Gauteng 0003, South Africa; School of Medicine, Faculty of Medicine and Health Sciences, University of Kinshasa, Kinshasa, Democratic Republic of the Congo; Department of Obstetrics and Gynaecology, Faculty of Medicine and Health Sciences, Walter Sisulu University, Private Bag X1, Mthatha 5100, Eastern Cape, South Africa; School of Medicine, Faculty of Medicine and Health Sciences, University of Kinshasa, Kinshasa, Democratic Republic of the Congo

**Keywords:** Democratic Republic of the Congo, e-SPAR, health security, International Health Regulations, NAPHS, One Health, outbreak preparedness, public health capacity

## Abstract

The Democratic Republic of the Congo (DRC) conducted a national self-assessment of the 2005 International Health Regulations (IHR) core capacities using the revised 2021 electronic State Party Self-Assessment Annual Reporting tool. Held in March 2022, the hybrid workshop involved multisectoral stakeholders and evaluated 15 core capacities. The average national score was 36%, indicating limited capacity to implement IHR and the 2019 National Action Plan for Health Security. Four capacities, including points of entry and radiation emergencies, showed no capacity, while 10 were classified as limited. Only the IHR coordination reached the developed capacity level. Key weaknesses were identified in emergency operations, laboratory systems and legal preparedness. Strengths included One Health integration and risk communication efforts. These findings underscore the urgent need to improve multisectoral coordination, operational readiness and infrastructure investment to meet IHR requirements and better respond to public health threats. Strategic recommendations were proposed to guide national and international support.

## Introduction

The International Health Regulations (IHR) of 2005 establish a legally binding global framework aimed at enhancing countries’ abilities to detect, assess, report and respond to public health risks that may cross international borders. All World Health Organization (WHO) member states have endorsed the IHR, which stresses the need for resilient national public health systems and incorporates a monitoring system via the electronic State Party Self-Assessment Annual Reporting (e-SPAR) tool.^1^ This tool allows countries to annually self-evaluate their capacities across 15 essential public health domains that are critical for global health security.

Despite the widespread adoption of the IHR, compliance has varied considerably worldwide. Wealthier nations such as the USA, Australia and various European Union countries generally report higher e-SPAR scores, largely due to robust legal frameworks, surveillance systems and healthcare infrastructure.^2^ However, even these countries experienced significant challenges during the coronavirus disease 2019 (COVID-19) crisis, particularly with risk communication, coordination efforts and supply chain resilience.^3,4^ In contrast, low- and middle- income countries often face persistent difficulties related to chronic underfunding, weak laboratory capabilities and limited multisectoral preparedness.^5^

The Democratic Republic of the Congo (DRC) has been repeatedly confronted by major public health emergencies, including numerous Ebola virus disease outbreaks, large measles epidemics and the COVID-19 pandemic. A Joint External Evaluation (JEE) conducted in 2018, which followed a 2017 self-assessment, reported an overall average IHR core capacity score of only 37%.^6^ In response to these challenges, the DRC launched a multisectoral National Action Plan for Health Security (NAPHS) in 2019 aimed at addressing these gaps and improving collaboration among the health, agriculture, transport and security sectors.^7^

To track progress since the JEE and evaluate how the NAPHS has been operationalized, a national workshop was held in Kongo Central Province in March 2022. This workshop applied the updated 2021 version of the e-SPAR tool and involved stakeholders from various sectors. The goals were to assess current capacities, analyse trends over time and identify priority areas for intervention. Early results indicated uneven progress, with some advances in coordination but persistent challenges in laboratory systems, points of entry (PoE) operations and emergency preparedness.

Although the DRC has made important strides through the implementation of the NAPHS, critical gaps remain in key IHR technical areas. Deficiencies in surveillance, laboratory infrastructure, border health security and cross-sectoral responses continue to undermine the country’s ability to effectively detect and manage emerging public health threats. Conducting a timely assessment using the revised e-SPAR tool is crucial not only for measuring status but also for guiding evidence-based updates to the national health security strategy and shaping donor and technical support priorities.^8^ Despite the implementation of the NAPHS in 2019, significant gaps remain in the DRC’s IHR core capacities, particularly in areas such as laboratory diagnostics, surveillance, risk communication and management of PoE, as suggested by the preliminary findings from the 2022 e-SPAR assessment. This study seeks to determine the extent of improvement in the DRC’s IHR core capacities since the 2018 JEE and to identify which specific capacities continue to be underdeveloped according to the latest 2022 self-assessment. The aims are to present initial results from the 2022 e-SPAR evaluation workshop, evaluate the progress made in implementing the 2019 NAPHS and highlight ongoing gaps to inform strategic priorities for both national action and international support.

## Methods

A hybrid national workshop was held from 3 to 9 March 2022 in Kongo Central Province, jointly facilitated by the DRC’s National Public Health Program (PNHF) and Directorate General for Disease Control, with technical and logistical support from the WHO and development partners. The updated 2021 e-SPAR tool was used to assess 15 IHR core capacities through 35 performance indicators. Each indicator was scored on a 5-point scale from 1 (no capacity) to 5 (sustainable capacity) and results were expressed as percentage averages, in line with the WHO’s monitoring framework.

The workshop included 60 participants representing multiple sectors—human health, animal health, agriculture, customs, environment and emergency services—participating both in person and virtually. Following the WHO’s One Health and multisectoral approaches, participants were divided into four thematic groups: prevention, detection, response and other IHR hazards (including chemical, radiological, zoonotic and PoE-related events). Each group independently reviewed and scored relevant indicators through facilitated discussions, reaching consensus before presenting their findings in plenary sessions for final validation.

Data from the workshop were analysed using descriptive statistics to calculate average scores for each core capacity and indicator, enabling comparison with prior self-assessments and the 2018 JEE. Qualitative feedback from group discussions was reviewed to contextualize quantitative results and identify operational challenges. This mixed-methods approach provided a comprehensive picture of the DRC’s IHR capacities, highlighting areas of progress and ongoing gaps to inform strategic priorities.

Ethical approval for the workshop and evaluation was obtained from the WHO and the National Department of Health of the DRC, in collaboration with PNHF. As the assessment was based on aggregated, non-identifiable data and expert consensus rather than individual-level research, formal individual consent was not required. Throughout the process, principles of confidentiality, transparency and voluntary participation were maintained, ensuring compliance with institutional and national ethical standards.

## Results

The 2022 e-SPAR self-assessment workshop in the DRC resulted in an overall IHR core capacity score of 36%, reflecting a limited level of national capacity in implementing the 2005 IHR and the NAPHS adopted in 2019. The assessment applied the WHO’s 2021 e-SPAR framework (second edition) to evaluate 15 core public health capacities, with scores assigned on a scale of 1–5 and expressed as percentage values.

Of the 15 capacities evaluated, 4 were classified as having ‘no capacity’ (score of 1 or <20%). These included PoE, food safety, zoonotic diseases surveillance and radiation emergency response operations, indicating severe weaknesses in hazard-specific preparedness and cross-sectoral systems. Ten capacities—national legislation, risk communication, laboratory services, surveillance, human resources, emergency framework, health services, infection prevention and control measures (IPC), finance and chemical events—were rated as having ‘limited capacity’ (score of 2, ranging from 27 to 53%), suggesting fragmented implementation and underperformance across foundational technical areas. Only one capacity, IHR coordination and national IHR focal point functions, was rated as ‘developed’, with a score of 60%, reflecting relatively stronger performance in coordination and reporting mechanisms. Table 1 presents a breakdown of each core capacity score, clearly illustrating the country’s most underdeveloped areas—particularly those linked to health emergency response operations and border health measures. Notably, several capacities, such as surveillance (30%), human resources (30%) and the national health emergency framework (27%), showed minimal improvement compared with previous evaluations, indicating stagnant progress in health systems strengthening.

**Table 1:** SPAR 2021 capacity scores—DRC (March 2022 evaluation).

No.	IHR core capacity (2021 SPAR, 2nd edition)	Score (1–5)	Score (%)	Capacity level
1	National legislation, policy, and financing	2	40	Limited capacity
2	IHR coordination and national IHR focal point functions	4	60	Developed capacity
3	Finance	2	30	Limited capacity
4	Laboratory capacity	2	44	Developed capacity
5	Surveillance	2	30	Limited capacity
6	Human resources	2	30	Limited capacity
7	National health emergency framework	2	27	Limited capacity
8	Health services	2	53	Developed capacity
9	Infection prevention and control measures	2	53	Developed capacity
10	Risk communication	2	40	Limited capacity
11	Points of entry	1	20	No capacity
12	Zoonotic diseases surveillance	1	50	Developed capacity
13	Food safety	1	20	No capacity
14	Chemical events		20	No capacity
15	Radiation emergency response operations	1	20	No capacity
	Average score	1.8	36	Limited capacity

**Figure 1:**
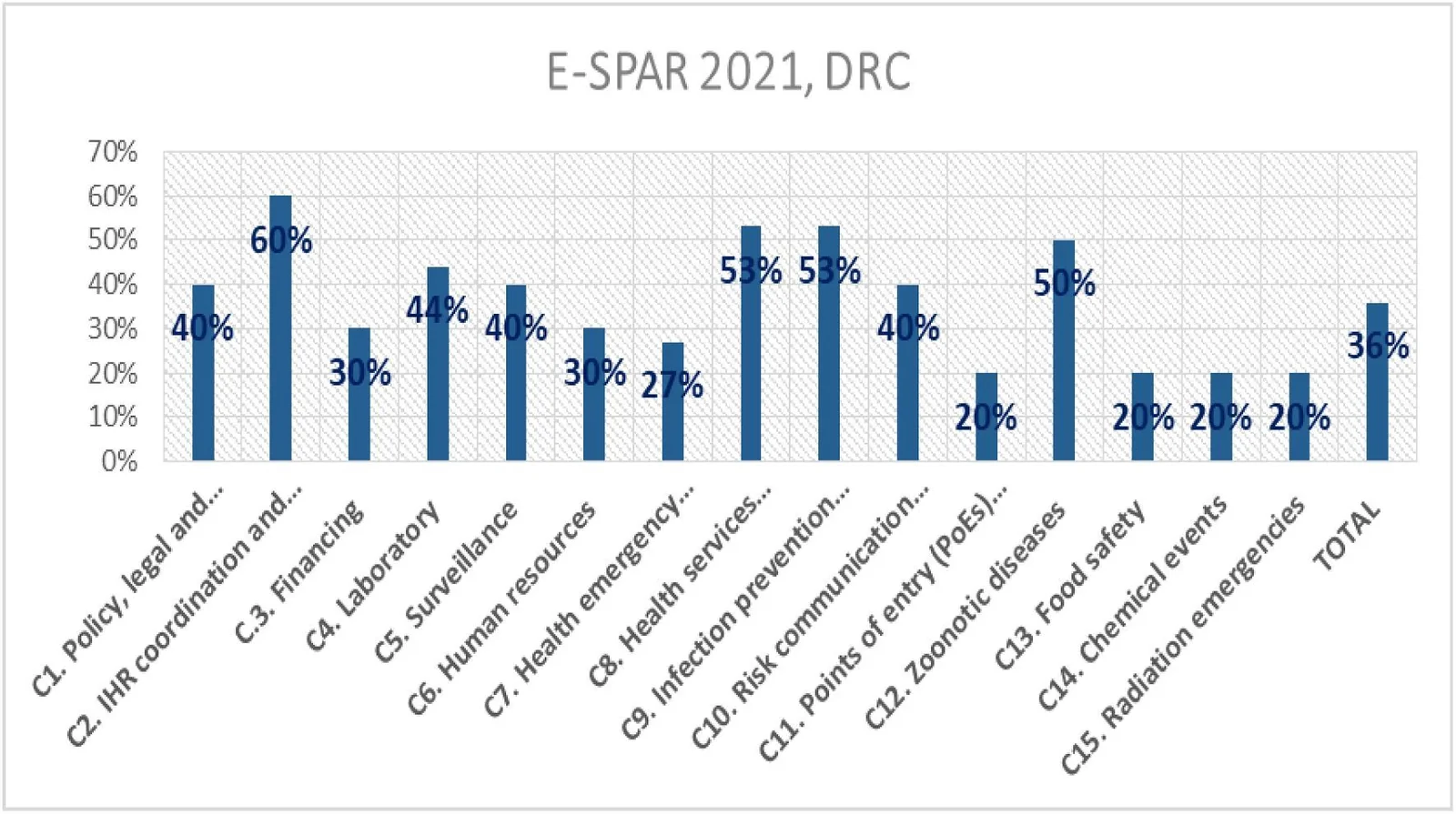
Score distribution by SPAR capability.

These findings highlight systemic issues in multisectoral coordination, legal frameworks and technical infrastructure that hinder the country’s ability to effectively detect, prevent and respond to public health threats. Figure 1 presents the distribution of scores across capacities, while Table 2 summarizes key strengths (such as One Health integration and emergency planning), limitations (e.g. legal gaps and weak coordination) and strategic priorities, including enhanced financing, intersectoral governance and legal reform for chemical and radiological preparedness. Overall, the results call for urgent investment and high-level commitment to reinforce the DRC’s core public health capacities in alignment with IHR requirements.

**Table 2:** Strengths, limitations, lessons learned and recommendations.

Category	Details
Strengths	Developed IHR coordination mechanism Ongoing IPC training Establishment of emergency plans (Ebola, zoonotic diseases) One Health integration has begun
Limitations	Poor multisectoral alignment Inadequate funding and emergency stockpiles Gaps in legal frameworks Weak surveillance and laboratory systems
Lessons learned	Need for real-time digital surveillance tools Risk communication must be culturally adapted Regular simulation exercises build readiness
Recommendations	Establish and fund national emergency response systems Strengthen laboratory networks at all levels Align partners around national priorities
	Fully implement the WHO IPC eight-component framework Improve vector control and food safety protocols Draft and enact laws for chemical/radiological events

## Discussion

This evaluation highlights persistent structural and operational challenges limiting the DRC’s full implementation of the IHR.^1^ Although political commitment and coordination have strengthened—evidenced by the NAPHS and active participation in global monitoring—the country’s overall capacity score of 36% reflects ongoing weaknesses across critical technical areas. This trend is consistent with many low- and middle-income African countries such as Nigeria, Ethiopia and Uganda, where progress toward IHR compliance remains uneven. In contrast, higher-income regions, including Europe and North America, generally report stronger IHR core capacities,^2,3^ although these regions still faced notable challenges during the COVID-19 pandemic, especially in risk communication and supply chain resilience.^4,5^

A positive development in the DRC is the gradual adoption of the One Health approach, fostering collaboration between human, animal and environmental health sectors. This is critical in a country where zoonotic diseases like Ebola frequently emerge. Similar One Health frameworks are gaining traction in African countries like Kenya and Tanzania,^6^ although full integration into routine operations remains a continent-wide challenge.

Significant gaps persist in laboratory capacity and disease surveillance, undermining outbreak detection and response. Such limitations are prevalent across many African nations, including South Sudan and Malawi, where diagnostic infrastructure and reporting systems are underdeveloped.^7,8^ This contrasts with more advanced laboratory networks in Asia, such as in Thailand and South Korea, and in Europe, where surveillance systems are better resourced and more timely.^9,10^

PoE preparedness remains a major vulnerability in the DRC, with limited contingency planning at airports and land borders. Similar weaknesses are reported in other African countries such as Cameroon and Chad, increasing the risk of cross-border disease transmission.^11^ Conversely, countries in the Americas and Europe have more established PoE management systems, contributing to better containment of infectious threats.^12,13^

Finally, inadequate emergency financing and outdated legal frameworks restrict the DRC’s responsiveness to health emergencies. This challenge is common across low-income countries globally, including parts of Asia and Africa.^14,15^ Addressing these governance and funding gaps requires sustained investment in health system strengthening, legal reform and institutionalization of emergency preparedness to enhance resilience against future public health threats.^1,7,16^

## Conclusions

The 2022 e-SPAR evaluation confirms that while the DRC has made modest gains in IHR implementation—particularly in coordination and emergency planning—critical operational gaps persist. A national capacity score of 36% indicates that most IHR core components remain underdeveloped. To advance health security, the country must prioritize laboratory strengthening, sustainable funding mechanisms, legal reforms and full operationalization of the One Health approach. Long-term resilience will depend on institutional commitment, effective multisectoral collaboration and continued support from international partners.

## Data Availability

All data generated or analyzed during this study are included in this published article. No additional datasets were used or are required for replication of the results.
